# 1528. Retrospective cohort study of factors associated with non-initiation of antiretroviral therapy among adults newly diagnosed with HIV in Andijan, Uzbekistan, 2018-2021

**DOI:** 10.1093/ofid/ofad500.1363

**Published:** 2023-11-27

**Authors:** Shokhruh Usmanov, Roberta Horth, Alfiya Denebayeva, Saya Gazezova, Sanam Zikriyarova, Botirjon Kurbanov, Feruza Nasirova, Dilyara Nabirova

**Affiliations:** Central Asia Field Epidemiology Training Program, Andizhan, Andijon, Uzbekistan; US Centers for Disease Control and Prevention, Dulles, Virginia; Almaty City Center for Prevention and Control of AIDS, HIV Center, Kazakhstan,, Almaty, Almaty, Kazakhstan; Central Asia Field Epidemiology Training Program, Andizhan, Andijon, Uzbekistan; Kazakh National Medical University named after S.D. Asfendiyarov;, Almaty, Almaty, Kazakhstan; Sanitary-Epidemiological Tranquility and Public Health Committee Tashkent, Uzbekistan, Tashkent, Toshkent, Uzbekistan; Andizhan State Medical Institute, Andizhan, Uzbekistan, Tashkent, Surkhondaryo, Uzbekistan; CDC Central Asia office, Almaty, Almaty, Kazakhstan

## Abstract

**Background:**

Immediate uptake of antiretroviral therapy (ART) by people newly diagnosed with HIV reduces morbidity and viral transmission. Since 2018, in Uzbekistan people newly diagnosed with HIV are immediately offered ARVs, but just over half of people living with HIV are estimated to be on ART. To reach global targets, we assessed factors associated with ART non-initiation.

**Methods:**

We conducted a retrospective cohort study using secondary data from Andijan Region Republican AIDS Center. Our study included all people 18 years of age and older newly diagnosed with HIV between January 1, 2018, and June 31, 2021. We analyzed sociodemographic and behavioral factors associated with non-initiation of ART, defined as not having initiated ART by December 31, 2021. Using multivariable analysis, we calculated risk ratios (RR) and 95% confidence intervals (CI).

**Results:**

From 2018 to 2021 in Andijan Province, 1,098 people were newly diagnosed with HIV, of which 113 (10.3%) did not initiate ART. Participants were mostly 30-49 years old (49%), male (56%), married (46%), and with secondary education (74%). Also, 39% and 31% had HIV clinical stage I and II, respectively. Risk for non-initiation was higher among people with secondary education (RR=8.6 [CI: 1.2–60.9]) compared to higher education, with multiple partners (RR=2.7 [1.5-5.0]), with disease stage I (RR=3.3 [2.0–5.2]) and stage II (RR=4.4 [2.8–7.0]). People with sexual partners living with HIV had higher risk of non-initiation (RR=1.8 [1.2–2.9]) than people with partners that did not have HIV. Being single was associated with reduced risk of non-initiation (RR=0.1 [0.03–0.3]).

Factors associated wtih initiation of ARV treatment among people newly diagnosed with HIV, Uzbekistan

**Figure:**
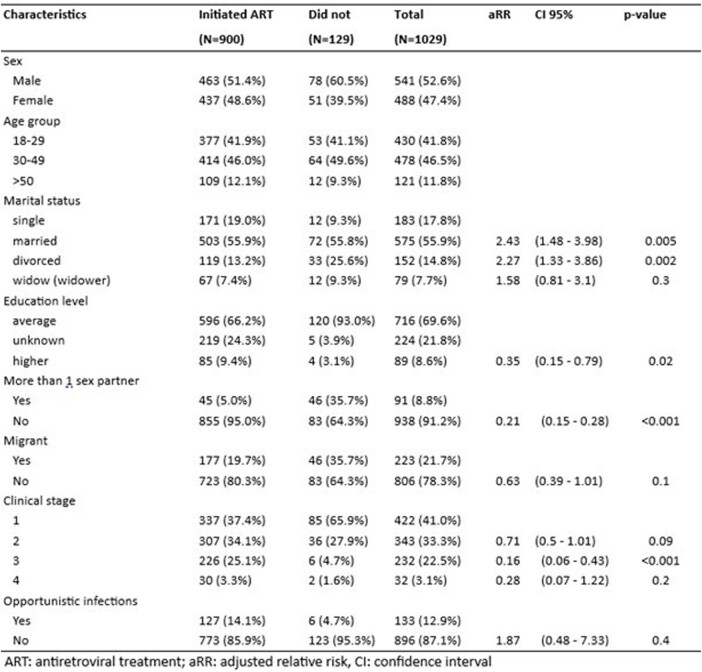

**Conclusion:**

People with multiple partners and with partners living with HIV should be prioritized for ART initiation support. Increased risk of non-initiation among people in non-symptomatic or mild disease stages earlier disease stages points to the need for interventions to increase awareness of test-and-start among providers.

**Disclosures:**

**All Authors**: No reported disclosures

